# Lost in Transition: The Dissemination of Digitization and the Challenges of Leading in the Military Educational Organization

**DOI:** 10.3389/fpsyg.2019.02049

**Published:** 2019-09-10

**Authors:** Torill Holth, Ole Boe

**Affiliations:** ^1^The Norwegian Military Academy, Oslo, Norway; ^2^Department of Business, Strategy and Political Science, USN School of Economics and Business, University of South-Eastern Norway, Drammen, Norway

**Keywords:** leadership, digital transformation, competencies, organizational behavior, development, military education

## Abstract

Leading change in adult educational organizations is not frequently described in the leadership literature. The education sector in the Norwegian Armed Forces (NAF) is in the middle of an educational reform that requires major changes. More students, fewer teachers and new organization of the studies as well as requirements for an increasingly updated technological expertise may mean that it will be necessary to increase the use of digital teaching aids. However, this is not systematically communicated as part of the reform in the same way as new topics of study. From a teacher’s perspective, the most important thing is to safeguard the quality of the education and ensure that important topics do not disappear in a reform. Therefore, one can well imagine that the focus on the purpose of change and the need for active participation is overlooked or not prioritized. Our focus in the process was largely the study content and some concern about the increase in the number of cadets. After completing the first courses in the new education, we were therefore quite surprised that the cadets were asked in the course evaluation whether digital aids had been used in the teaching, As a result, we were inspired to look more closely at what requirements were set and whether more exact plans had been prepared for the introduction of digitization of education as part of the reform process. Since the education reform in the NAF results in such a fundamental change, our perspectives may possibly benefit staffs at other colleges who are going to carry out major change processes. The main goal of this study was to investigate if or how the Norwegian Ministry of Education and Research’s intention of digitization and its specific primary goals of learning and teaching trickle down through the hierarchies and into the study programs at the Norwegian Military Academy (NMA). To see if the Ministry’s intentions were actually understood and realized, as our second aim we investigated whether we found the concepts of digitization or digital tools mentioned in any of the Norwegian Defence University College’s (NDUC) study programs and subject plans for teaching. These intentions cannot be implemented unless they are enshrined in the study programs. As a third aim we also tried to find out whether digitization and digital tools actually had been used in the teaching in the new NAF Basic Officer Education, as this would reflect how the Ministry’s intention of digitization and specific primary goals of learning and teaching had been realized. We used a mixed methods approach in the study as we first investigated documents compiled from the government issued for the university and college sector in Norway, the NAF and the NDUC to see if the overall plan for digitization from the Norwegian Ministry of Education and Research could be traced. In addition, we investigated the answers that officer cadets had given to questions in three course evaluations related to the use of digital tools in their education. Our three hypotheses were the following: Hypothesis 1: Several of the requirements for digitization have disappeared in the dissemination of the documents from the Ministry to the NAF Basic Officer Education. Hypothesis 2: No plan has been prepared as part of the educational change process for the introduction and implementation of new digital tools in the NAF Basic Officer Education. Hypothesis 3: It is up to the individuals to introduce and implement the use of new digital tools in the NAF Basic Officer Education. We found support for the two first out of our three hypotheses. The latter proved difficult to investigate with the means available, but we will nevertheless discuss some assumptions we have formed, based on the findings that the survey revealed. Hypothesis 3 only received partial support. Finally, the article discusses some leadership challenges that arise from the results we found. The study thus shows how long it can take from the Ministry issues its intention until it is actually implemented in practice at the NDUC. The final comments may also give an indication of how this process may be better focused and thus become likely to increase the implementation rate of digitization.

## Introduction

“*The future cannot be predicted, but futures can be invented*.”

(Gabor, 1963)

The Norwegian Armed Forces (NAF) is under a certain political pressure to become a modern competence organization (Heier, 2017). The military education should be coordinated, modernized, streamlined and cost-effective including education of both Operative Officers and Engineer Officers (OF) and Other Ranks (OR) (Forsvarsdepartementet, 2016b). Good quality should be achieved by using fewer resources. As the process of change in the education sector in the NAF is the result of statutory orders and thus not an internal desire to create improvements in education (Dokter and Aspelund, 2017), our purpose in this study was to investigate whether or not the goals stated by the Norwegian Ministry of Education and Research (Kunnskapsdepartementet, 2017a) regarding digitization, learning and teaching could be rediscovered in practical study programs in the military education. Digitization can be seen as the strategic use of technology in the educational context in order to facilitate learning and to create flexible learning systems (Elstad and Hafnor, 2017). However, we considered that there was a probability that they simply became lost in transition while being disseminated through the educational system. This was interesting because the NAF comprehensive education reform could require digitization of learning materials, teaching methods and communication.

In order to carry out such a major education reform, a change oriented leadership is required to manage the change process. At the same time, it is difficult to predict all the effects such a reform can bring. Change management is more a matter of continuous development and adaptation than a defined change process (Yukl and Lepsinger, 2004). A persistent ability to adapt to new trends and environments is required of organizations to remain competitive, and yet numerous studies show that the vast majority of change processes do not succeed. Dobrovič and Timková (2017) have included studies of Beer and Nohria (2000) and Decker et al. (2012) which suggests that as many as 70–93% of all change processes fail. The prerequisites for NAF to succeed in implementing digitization as part of the transition should have been reasonably satisfactory as the organization has a conscious attitude to technology as an important aid to creating operational capacity. The NAF is an organization that for several years already has introduced digital systems that allow top management to follow operations from a distance and even have the ability to directly influence and intervene in the events while they are ongoing (Forsvarsstaben, 2014). On the other hand, research has shown that even a highly digitized part of the NAF, the NAF Cyber Defense, struggles with strategic competence leadership, organizational structures, competence development, needs, plans, handling and communication (Boe and Torgersen, 2018). The Norwegian military education has not been exposed to the very big upheavals until now.

The change process in the NAF was planned at the same time as the Ministry of Education and Research launched its strategy on digitization of higher education “Digitization Strategy for the University and Higher Education 2017–2021” (Kunnskapsdepartementet, 2017a) and joining these processes together could possibly create a real change in both form and content and enable the work toward the modernized education that is needed.

The strategy plan states that digitization is supposed to help achieve educational goals and research in a better and more efficient way and contribute to interaction, raise the quality and relevance of research and higher education, and be tools that contribute to a more efficient, solid and well-functioning higher education and research sector. The digitization strategy is binding for the three Norwegian military academies, namely the Norwegian Military Academy (NMA) (Army), the Royal Naval Academy and the Royal Air Force Academy, and for the Norwegian Defence University College (NDUC) as a whole, as the Norwegian military academies are administratively organized under the NDUC. Digitization is supposed to help achieve educational goals and research in a better and more efficient way. As such, digitization is not an end itself. Implementing digitization will require anchoring ideas in the management, as well as organizational development and cultural change (Kunnskapsdepartementet, 2017a).

The main object of the reform process was to improve the NAF’s ability to meet the government’s quality reform goals within the higher education sector in general (Kunnskapsdepartementet, 2014–2015). The process should also fulfill a goal of creating robust professional groups (Forsvarsdepartementet, 2016b). At the same time, the education reform was intended to save costs by reducing the number of teachers and staff members to allocate more funds to the operational part of the organization.

After presenting the study’s objectives and hypotheses, the article seeks to create a holistic insight into how an intention appears to be realized by describing the Ministry’s expectations and then studying how these are answered in the NAF’s own documents. We have therefore divided the article into chapters that first describe the Ministry’s intention, before we describe the current situation in both Norwegian higher education in general and military education in particular. Then we try to comment on the Norwegian Ministry of Education and Research’s intended end state and see this in connection with NAF’s strategic investment in digitization. We have also examined some of the central goals that the Ministry has drawn up and assessed these against the need for an organizational and cultural change process. We conclude by making some suggestions for necessary leadership to approach national objects. In this way, we try to create coherence in the article by discussing intention, present situation, future vision and goals as well as challenges for the implementation process.

## Aims of the Present Study and the Hypotheses

In particular, our aim was to investigate the extent to which the NAF and the NDUC managed to cope with the intention of digitization and the introduction and implementation of digital tools as stated by Norwegian government (Kunnskapsdepartementet, 2017a).

The NAF have a long tradition of educating their own soldiers and officers (Hosar, 2000), and there are clearly strong opinions on how to learn the profession and what the education should encompass. Somewhat less focus has been on renewing learning methods until recently.

Based upon the previous presentation of the challenges related to the education reform and the transition process, the requirements for digitization and the need for leadership related to the changes that were to be implemented, we set out to investigate the following hypotheses:

Hypothesis 1: Several of the requirements for digitization have disappeared in the dissemination of the documents from the Ministry to the NAF Basic Officer Education.
Hypothesis 2: No plan has been prepared as part of the educational change process for the introduction and implementation of new digital tools in the NAF Basic Officer Education.
Hypothesis 3: It is up to the individuals to introduce and implement the use of new digital tools in the NAF Basic Officer Education.

In the present study, we emphasize a conceptual approach that we have based upon Glaser and Strauss grounded theory (1967). In addition to this, we have also used a document study to review the governance documents related to digitization of the educational sector in Norway and documents that also applies to the entire NAF. As such, the present study thus reveals an educational-psychological *status quo* regarding digitization for the new Basic Officer Education in Norway. An investigation of the educational-psychological *status quo* within the Basic Officer Education will also describe the current mental state within the Norwegian military educational sector. If the current mental state also reveals that the NDUC prefers the current status, this might present a barrier to the NDUC as the leaders and especially the teachers might prefer to stay with what they already know, may cause challenges when it comes to leading in a more digitized and complex educational environment. This barrier may hamper the possibilities of developing new measures to deal with the introduction and implementation of digitization, and may in the end lead to a less effective military educational system.

## The Norwegian Ministry of Education and Research’s Intention of Digitization

In recognition of the fact that digitization is changing much of our everyday life, the Norwegian government initiated the design of a digitization strategy for the university and college sector. Well thought-out and well-structured digitization strategies should ensure good connection between technology and organizational and practice changes at all levels. Both institutions and administrative agencies are encouraged to develop their own digitization strategies for using technology for learning and research. There is a need for the Ministry of Education and Research to provide an overall strategic direction for the higher education sector’s work on digitization through clear expectations and future projections, and clarification of the task and responsibility distribution (Kunnskapsdepartementet, 2017a). The national digitization strategy has a four-part goal. Table 1 below gives an overview over these four goals.

**TABLE 1:** The four goals of the national digitization strategy (p. 9, authors’ translation).

(1) High quality in education and research. (2) Research and education for welfare, value creation and restructuring. (3) Good access to education. (4) Effective, diverse and solid higher education sector and research system.

This is supposed to increase student learning, make higher education more accessible to more students, and support outstanding research. The Ministry of Education and Research points out that if this is to be achieved, the work must be raised to a strategic level at every single institution and integrated into all academic and administrative activities (Kunnskapsdepartementet, 2017a). This will require that the academic communities at the various educational institutions reflect on how effective teaching methods based on research on learning and development can be used to create good learning and variety in assessment methods (Kunnskapsdepartementet, 2016). The Norwegian government expects the professional communities to use teaching methods where students are active in their own learning process and that they use digital aids and new technologies where appropriate and possible (Kunnskapsdepartementet, 2017a).

The overall digitization strategy should be operationalized through follow-up of the sub-strategies in the areas of research, education, infrastructure, administrative solutions and information security. Each institution is expected to manage its own digitization work through own goals and strategies, which are to be adapted to the sub-strategies and the overall digitization strategy. A high degree of local initiative and follow-up is thus required. It is therefore difficult to assess whether today’s handling of the instructions is in accordance with the expectations provided in the governing documents.

In this article, we mainly concentrate on the first goal of the national digitization strategy, namely high quality in education and research, and especially education.

## The Current Situation in Higher Education in Norway

The report *Concentration for quality – Structural reform in the university and college sector 2014–2015* published by the Norwegian Ministry of Education and Research defined as one of its goals to increase the quality of research and education by gathering smaller campuses under a joint organization (Kunnskapsdepartementet, 2014–2015). The main goal of the structural reform is universities and colleges of high quality in education and research and robust professional communities where the resources are used as much as possible on the core tasks, education and research (Kunnskapsdepartementet, 2014–2015). The report states that in a future labor market, a higher level of competence is required. There will be a need for practitioners who can exercise ethical reflection, creative problem solving and critical thinking. They must be able to handle complex and ambiguous information and collaborate across geographical, professional, and cultural boundaries. Table 2 reveals the Norwegian government’s definition of quality in higher education.

**TABLE 2:** The Norwegian government’s definition of quality in higher education (p. 15, authors’ translation).

Quality in higher education is that students should: (1) Achieve the best possible learning outcomes and personal development (2) Encounter relevant education that prepares them well for active participation in a diverse and democratic society and for a future professional career (3) Achieve the education as effectively as possible.

These ambitions can be gathered under the heading “learning outcomes, relevance and implementation”. Surveys from 2014, conducted by the Norwegian Agency for International Cooperation and Quality Enhancement in Higher Education, reveal a challenge in that digitization within the university and college sector in Norway so far largely has been linked to personal initiative and interest (DIKU, 2014).

The Regulations on the National Qualifications Framework for Lifelong Learning with its reference to the European Qualifications Framework for Lifelong Learning (Kunnskapsdepartementet, 2017b) state that it is the responsibility of the Norwegian Ministry of Education and Research to implement the National Qualifications Framework within the university and college sector in Norway. The three Norwegian military academies as well as the overarching NDUC are, as members of the university and college sector in Norway, also bound by these regulations.

The aim of the work on qualification frameworks is to make the education systems more understandable both nationally and internationally, to facilitate increasing mobility within and between countries, and to contribute to flexible learning pathways and thereby strengthen lifelong learning. The Ministry’s understanding of a national qualification framework is formulated as follows: “A qualification framework is an overall, systematic and level-divided description of formal qualifications that can be achieved within an education system. The framework is a systematic description of the level and competence acquired for the levels in the Norwegian education system. National qualifications frameworks are based on the nation’s education system” (Kunnskapsdepartementet, 2011, p. 7).

Several institutions have adopted their own strategies for digitization or incorporated targets for digitization in new institutional strategies. Digitization is increasingly being linked to quality of education. There has been particularly high activity with regard to digitization of the exam (Kunnskapsdepartementet, 2017a).

The academic staff in the university- and college sector have called for competence enhancement and support functions for the use of digital tools. Stronger management support, more joint solutions and more efficient management and organization have also been called for Kunnskapsdepartementet (2017a).

Many tasks are still carried out analogously at the individual educational institutions. Digital tools for administration and assessment has been used to a greater extent than for learning and research in the university and university college sector (Kunnskapsdepartementet, 2017a).

Surveys conducted in 2018 by the Norwegian Agency for International Cooperation and Quality Enhancement in Higher Education (DIKU, 2018), highlight that few applications sent from universities and colleges to the Norwegian University in 2018 focus on the technology itself. Instead, most of them describe projects that will solve academic or educational and didactic challenges. The applications show a clearer link between digitization and educational quality than before. The technology creates new conditions for student participation. The agency states that this reflects a maturity in the organizations’ use of technology and their willingness to change and develop (DIKU, 2018).

The agency refers in its report series to potential strong links between digitization and educational quality. However, a systematic cooperation stretching from central authorities to local operational level is required. The agency points out that the development of teaching quality requires collaboration between a number of actors at different levels from national authorities via managers at the educational institutions and right down to the individual teacher in a ‘chain’ where quality must exist at all levels (DIKU, 2017).

## The Current Educational Situation in the NAF

In the summer of 2016, the Ministry of Defense announced that it needed a reform of the education system in the NAF. The education system was considered to be suffering from fragmentation, with small competence clusters and complex management lines. The goal of the reform was to provide better conditions for creating a high-quality and flexible education system, which also is cost-effective. One common new college for level-forming education was thought to facilitate more robust academic communities and strengthen the research-based education (Forsvarsdepartementet, 2017). From January 2018, the military academies were merged into a joint college in line with the Ministry’s structural requirements, however retaining their original names and locations. They also kept their own college director, while the staff were rationalized and functions were centralized. In practice, the education reform was implemented from 01.08.2018. From the same date the new cadets were supposed to be taught according to the new curriculum and have more common teaching located at the NMA.

However, the qualification framework with the design of learning outcomes is well known in the military education sector. Admittedly, there are, in part, major differences in how the different schools design the learning outcomes and what is emphasized. Digitization has been reflected to a small extent in the different learning outcomes.

In the summer of 2016, the Norwegian Defense Research Establishment (NDRE), in collaboration with the NDUC, conducted a survey among employees and students at the NAF schools. The aim was to gain insight into how systematically the schools approached digitization in teaching (Elstad and Hafnor, 2017).

The NDRE considered it necessary for the schools to start thinking systematically about digitization in teaching in order to prepare for the upcoming educational reform, and prepared a full report that could serve as a framework for further strategic and systematic work on the digital development for the new Basic Officer Education in the NAF. The report notes that there are three critical factors for the new organization to be able to move from individual enthusiastic teacher practice to strategic venture. These factors are *leadership and organizational change, digital competence*, and *structured flexibility* (Elstad and Hafnor, 2017). This means that the management group must have consensus on a strategic perspective of the digitization process, and what opportunities digitization in the teaching should provide. Digital competence is a critical resource and the employees need both knowledge and skills with digital teaching aids, and they need to be able to see the usefulness of the technology in the education. This competence also includes the employees’ willingness to explore and apply new teaching methods. In order to make this happen, the report also suggests that incentives should be available for the teaching staff who are innovative and adopt a wider range of digital solutions.

The academic communities that were described as small have become even smaller as a result of the education reform. In this way, the military academies are becoming a larger entity organizationally, but due to the fact that they are still located around the country, the academic communities do not initially become more robust. The teaching is, however, expected to take place jointly in parts of the education course. This requires changing and developing the schools’ thinking about their teaching for the cadets to achieve the learning outcomes and personal development.

For the Norwegian military education sector, there is as yet no systematic effort to increase the digital competence of the teaching staff. Digital tools are widely used for study planning and exams, but until recently different teacher and student groups at each military academy have used several different tools simultaneously.

After the restructuring, no incentives have been introduced to implement digital learning spaces and learning platforms. There is almost no time allocated to research and development after the transition, which included a noticeable downsizing. There are several different digital tools that the NDUC wants the teachers to use and there is occasional introduction courses to how to use them. There is a great willingness to help but no systematic effort.

## The Norwegian Ministry of Education and Research’s Intended End State

The Ministry of Education and Research defines overall goals and desired direction for the new proposed digital venture (Kunnskapsdepartementet, 2017a). The digitization strategy clarifies the organization of the higher education and promotes actions that enable the sector to respond quickly to opportunities and challenges using ICT. ICT is an abbreviation of information and communications technology. Digitization in the NDUC’s education thus requires some caution with regard to the protection of personal data. The Ministry expects that opportunities for new and changed learning and teaching processes and new organizational and communication forms will arise through digitization. The use of learning analysis, including understanding students’ learning patterns and improving learning processes, is barely in an initial phase. The overall objectives of the university and college sector are given in the Norwegian government proposition 1S (2016–2017) which says that the government invests in research infrastructure to have world-leading professional communities. This is recently confirmed in the long-term plan for the period 2019–2028, where the three overall goals for research and higher education are stated (Kunnskapsdepartementet, 2018) (see Table 3).

**TABLE 3:** Overall goals for research and higher education (p. 7, authors’ translation).

(1) Strengthened competitiveness and innovativeness (2) Meeting major social challenges (3) Developing professional communities of outstanding quality

The main objective of the Ministry of Education and Research’s digitization strategy is that digitization should contribute to achieving the main goals of the sector (Kunnskapsdepartementet, 2017a).

The Ministry of Education and Research (Kunnskapsdepartementet, 2017a) has prepared goals for six different perspectives or groups. Goals can be described as internal representations of desired states or outcomes (Grant, 2012). The goals represent a direction that will govern the development of sub-strategies and the implementation of measures in each of their areas (Kunnskapsdepartementet, 2017a). The goals have been prepared for the student, the teacher, the researcher, the management at all levels, for data and infrastructure, and for administrative systems. In this article, we will primarily focus on the goals set for the teachers, who are those who develops the new military education, as well as looking at the goals related to leaders in management positions.

## The NAF’s Intended Future Educational Situation

In September 2018, the NAF digitization strategy was launched (Forsvarsstaben, 2018). The Chief of Defense states in his preface that the NAF must change in line with the surrounding world. He states that: “Around us we see that technological developments are progressing faster, and in the years to come I believe this will lead to major changes in the way we work” (p. 2, author’s translation). Table 4 reveals the five priority areas in the digitization strategy.

**TABLE 4:** Areas prioritized in the NAF digitization strategy (p. 3, authors’ translation).

Management model and architecture
Smart systems
New digital interaction
Information security
Digital competence.

In order for the NAF to reach its digital ambition, efforts must be made within the five strategic priority areas that match one or more of the digitization goals. In this context, we only mention what the plan prioritizes under the focus area of digital competence. The most obvious strategic goal the NAF’s education sector can focus on is this one: NAF’s’ competence and culture must be adapted to a digital everyday life.

The strategy states clearly that restructuring must be anchored and led from the top of the organization. The management must set a course that develops the organization. The plan confirms that: “To see digital technology from a strategic perspective, the NAF must first and foremost understand how the whole organization should work with innovation and learning.” (Forsvarsstaben, 2018, p. 27, authors’ translation).

Three objectives have been outlined with underlying measures and we mention two of those we believe are the most relevant to this article. Table 5 outlines the objectives found in the NAF digitization strategy.

**TABLE 5:** Objectives outlined in the NAF digitization strategy (p. 29, authors’ translation).

(1) The art of making mistakes quickly. One of the measures states that the NAF must provide for a cultural change. Failure is allowed as long as we learn from our mistakes. (2) Digital leadership, where two of three measures are: (a) Change management and employee development and (b) raising competence at management level.

The NAF’s digital competence should be improved by developing ambassadors for digitization and creating a culture that promotes innovation and digitization.

This strategy is designed to see the NAF in the large context, and not specifically to design the NAFs’ education system. The operating units are responsible for developing their own strategies to meet the overall objective – that of a flexible defense with better responsiveness and closer cooperation with national and international collaborators, within the five priority areas and two main pillars: increased operational ability and increased efficiency through digitization.

The strategy document also mentions the strategy as a digital transformation. It is interesting because it can be compared to what the Chief of Defense says about how digitization will change our way of organizing and working in the future. Many analyses have been carried out in the preparation of the strategy, and the internal analysis from the NAF reveals that the organization has low maturity in terms of digital competence (Forsvarsstaben, 2018).

## The Norwegian Ministry of Education and Research’s Specific Primary Goals of Learning and Teaching

Learning can be defined as a subjective process that occurs through activity and reflection in the meeting between students and teachers (Kunnskapsdepartementet, 2016).

As a teacher, one has through the Act on Universities and Colleges an independent academic responsibility for the content and structure of teaching within the framework established by the institution (Kunnskapsdepartementet, 2005). Table 6 reveals the goals that are set for the teacher in the digitization strategy for the higher education sector 2017–2021 (Kunnskapsdepartementet, 2017a).

**TABLE 6:** Goals set for the teachers in the digitization strategy (p. 10, authors’ translation).

(1) The teacher has a good digital and pedagogical competence (knowledge of how to use digital tools to promote learning in his or her subject), incentives for academic/pedagogical development of own teaching and access to collegial communities and support services for the development of study programs and sharing of digital learning resources. (2) The teacher has a wide range of applications and digital tools and services that support the implementation of the education, from planning through the implementation of teaching and interaction with students and colleagues, internally and externally, to follow-up and assessment of students at the individual and group level. (3) The teacher has opportunities for gains (in the form of promotion, qualification, salary), or time to develop the educational activity on the basis of documented results in the educational field.

Surveys made for the Ministry of Education and Research uncovered that the academic staff in the university- and college sector called for competence enhancement and support functions for the use of digital tools when the strategy was compiled.

In the report *digital state 1/2018, Digitization for quality of education and active learning in higher education* (DIKU, 2018), it now turns out that several of the educational institutions have established a professional community that “through relevant and partly research-based use of digitization both transcends and transforms established teaching practices.” (p. 5, authors’ translation). Digital technologies are no longer referred to as “tools” but are deployed in innovative projects and strategies to develop and renew educational practices and learning designs. On the other hand, it may seem as if knowledge about what characterizes active learning is somewhat weaker as it is not frequently described in detail.

Table 7 displays the goals that are set for the leadership at all levels from the same digitization strategy (Kunnskapsdepartementet, 2017a).

**TABLE 7:** Goals set for the leaders at all levels in the digitization strategy (p. 11, authors’ translation).

(1) The management utilizes the opportunities that digitization provides to achieve the institution’s goals by including digitization both in planning and in concrete measures and processes. (2) The management is conscious of its leadership responsibility and has the competence to lead, motivate and support necessary change processes as due to digitization. (3) The management utilizes the opportunities digitization provides to streamline administrative support functions and ensure good management. (4) The management safeguards the institution’s values and interests, and follows national guidelines through systematic efforts to strengthen information security. (5) The management ensures formalized systems for documentation of, and reward for, work on the development of teaching. (6) The management sets the level of ambition and facilitates for the entire academic community, not just enthusiasts, to use the opportunities digitization provides to raise the quality of education. (7) The management ensures that systems that are chosen make it possible for interaction within the university and college sector and with actors outside the university and college sector. (8) The management has good access to information and decision support.

The Norwegian Agency for International Cooperation and Quality Enhancement in Higher Education report 1/2018 (DIKU, 2018) also shows results in relation to management anchoring and more systematic and targeted management of the digitization work. One now sees that digitization is part of a more comprehensive quality work involving the institutions’ management, with more clear strategic anchorage and management than has been the case previously.

## The Norwegian Ministry of Education and Research Gap Analysis – How to Get There

Each institution is expected to steer their own digitization work through their own goals and strategies adapted to the sub-strategies and the overall digitization strategy. The measures are described in the Ministry of Education and Research’s ICT strategy (Kunnskapsdepartementet, 2017c) with holistic solutions in the Norwegian university and college sector specifically aimed at enhancing competence in teaching. In particular, it focuses on measures to increase the digital competence of the teachers to enable them to carry out the desired restructuring of the education in line with what the goals describe (Table 8). This change process will also place greater demands on management roles and good support functions.

**TABLE 8:** Measures described in the Ministry of Education and Research’s ICT strategy (p. 13, authors’ translation).

(1) Focusing on education management at all levels of the management apparatus related to the implementation of education. Professional leaders at all levels must be made aware of their responsibility for digitization of education. (2) The institutions must establish sufficiently powerful communities with the important combined pedagogical/didactic/technological competence necessary to support the academic environments in renewing the learning processes. Funding for educational development must be developed at the individual institution, and not least under the auspices of the proposed competition arena, which is meant to stimulate the renewal of educational processes. (3) The institutions must, to a greater extent than today, make the development of teaching and education meritorious. (4) In order to improve the digital competence of the next generation of students, education for digital competence must be strengthened and made compulsory in all Norwegian teacher education at all levels.

Universities and colleges are responsible for the quality of their education programs. The digitization strategy reaches forward to describe a desired state in the future. Table 9 points out the goals set in the strategy to achieve a desired situation (Kunnskapsdepartementet, 2017a). The measures in the strategy should contribute to promoting digitization as an instrument in the institutions’ work on quality of education. In this context, we have selected those that are specifically aimed at our research area.

**TABLE 9:** Goals set in the digitization strategy aimed at getting to the desired situation (p. 14–15, authors’ translation).

(1) Strengthen research on the connection between quality and changed learning processes based on digitization. (2) Universities and colleges define goals and concrete measures related to the digitization of learning processes and the use of new forms of learning to raise the quality of higher education. (3) Requirements for basic pedagogical competence and teaching experience when hiring in all academic positions, and successively higher requirements for teaching competence for appointment to higher-level positions. (4) Requirements for qualification systems for educational expertise and pedagogical development work at all institutions. (5) Strengthen the teachers’ digital competence to carry out the restructuring and further development of learning processes based on the new possibilities that digitization can give.

The goals that can be found in Table 9 thus give an indication of how the Ministry of Education and Research (Kunnskapsdepartementet, 2017a) thinks when it comes to reducing the gap between the current situation regarding digitization and how to get to get to the wanted situation.

## Leadership Challenges and Educational Implications in the Military Educational Sector

This part of the article takes into consideration how the military education sector manages to meet the goals set by The Ministry of Education and Research, as mentioned above. Leadership has been considered one of the most important components in the success of organizations (Landis et al., 2015). Leadership in the military can be explained as the process that creates a common direction, alignment and commitment in a military unit (Boe et al., 2015, 2017). It has been stated that no degree of technological development or scientific calculation will overcome the human dimension in war (US Marine Corps, 1989). The modern operational military environment is a mix of different factors. These situations are also referred to as VUCA situations, an acronym used to describe the volatility, uncertainty, complexity and ambiguity of different conditions and situations (Stiehm, 2002). The security threats that the military forces are facing in the 21st century bear the hallmarks of being multidimensional, transnational, and very often unpredictable (Fun and Ang, 2017). Advanced technologies will influence how individuals and groups collaborate, how they communicate, and how they engage with each other. They will also influence how individuals interact with the technology itself.

New technologies are being harnessed to improve leader and team development for greater effectiveness in operational environments (Do et al., 2017). While technology can be a solution to the problems faced by militaries, it can also be its own problem in need of a solution (Cassivi, 2017). Augier and Guo (2017) point out that although aspects of the technology are important, the human dimension of leaders and managers will still continue to play important roles when it comes to identifying new technologies, and in translating these technologies into new strategic opportunities. Furthermore, Augier and Guo state that factors such as organizational structures, routines and capabilities will also be needed, to support both the emergence and implementation of innovative technologies.

The term VUCA may represent the ongoing change processes to some extent, although not in any way comparable to the situations the forces encounter in their operations. Change processes are also often characterized by complexity, uncertainty and ambiguity in a way. Since resistance to change is common in organizations, explaining why change is needed is a key leadership behavior during organizational change (Yukl, 2012). The education reform in the NAF is initiated by the government to modernize, build stronger professional communities, and cut costs. This implies that the change basically is not defined as necessary by the teachers or the leadership in each military academy. This may make it even harder for the leaders to implement the required behavior by the subordinates to operationalize the intended goals, even though they are fully aware of their responsibilities. Looking at the goals for teachers in Table 6, they might seem a bit overambitious to achieve for the NAF in the period up to 2021 due to the major transition that the education sector is just about to implement. At the time of writing, no strategy has been drawn up for digitization in the education at NAF.

To implement an innovative strategy may fail due to resistance to change by members of the organization. A major change, like changing the teaching habits and introducing new technology, may elicit strong emotions. Resistance to change is more likely to occur when the employees do not agree to or understand the need for change (Yukl and Lepsinger, 2004).

At the same time, the teaching staff are aware of the role of technology in various military operations and therefore also see the need to make this a natural part of the teaching. Digitization is currently mostly used in the administrative functions, but in recent years it has also begun to make its entry into the pedagogics. On the personal level, a comprehensive process of change will reshape the social balance between the employees and the perceived beliefs of the individuals own mastery. Learning new ways of doing well-known tasks can require a lot of effort by some individuals, while others easily master and achieve new positions in the community (Yukl and Lepsinger, 2004). Skills that were previously considered important can quickly appear to be superfluous or unnecessary and old-fashioned.

**FIGURE 1:**
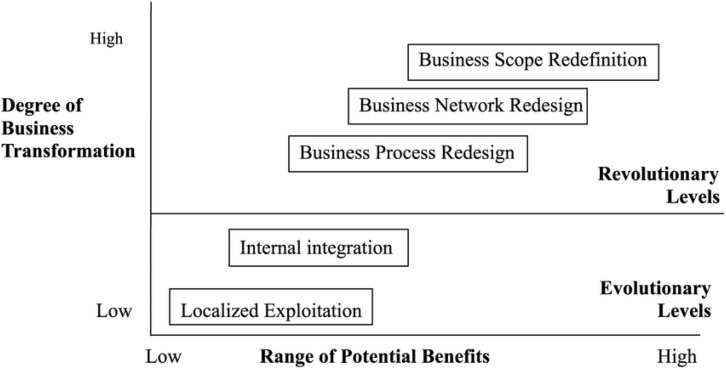
Venkatraman’s (1994) model of five levels of IT-enabled business transformation.

The report from the NDRE refers to a model developed by Venkatraman in 1994 (Elstad and Hafnor, 2017, p. 11). Figure 1 illustrates that gain realization by digitization is in a linear relationship to the degree of conversion in the organization. The biggest gain realization therefore necessitates the greatest organizational change (Venkatraman, 1994).

The two lowest levels in the model indicate an evolutionary process where the organization continues to do the same tasks, but supported by technology. This means that the work processes are only changed to a limited extent, and this may mean that the desired result cannot be realized. For the new military education it may mean that the teaching staff’s ambitions are not adjusted, or there are fewer people who perform the same tasks that were previously distributed among several (Elstad and Hafnor, 2017).

It can also reduce the possibility of realizing the development of the desired robust professional clusters. The process will be gradual and will require an attentive, targeted change leadership where the staff are involved in the need for change and lead the development of an effective strategy for achieving a mutually agreed upon future outcome.

**FIGURE 2:**
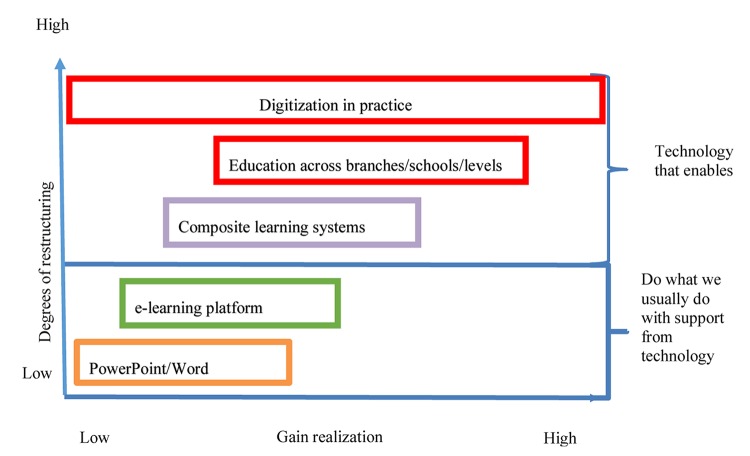
A new window for learning (Elstad and Hafnor, 2017, p. 25, authors’ translation).

Elstad and Hafnor (2017) describe a model for how to achieve a new window of learning. They claim that the schools are able to look at technology as an enabler for new and better forms of teaching. The goal must therefore be that employees automatically use technology that is more targeted and directed toward achieving gains. This, however, requires that the digitization dimensions are included as a seamlessly integrated part, both in terms of structure and process, at the new military education. Figure 2 reveals the model.

The figure can be explained by the two axes. The *x*-axis describes Gain realization and the *y*-axis illustrates the Degrees of restructuring. Both Gain realization and the Degrees of restructuring go from low to high. The highest level describes Digitization in practice. In descending order, the levels describe respectively Education across branches/schools/levels, and Composite learning systems (Elstad et al., 2017). These levels consist of technology that enables a high Degree of restructuring and also result in a high level of Gain realization, The two lowest levels, e-learning platform and PowerPoint/Word are levels where technology simply allows one to continue doing what one already is doing, with the support of technology. A low to medium effect of Gain realization can be seen here. Also a low degree of Restructuring will result of staying in these two levels. Stated differently, the two lowest levels gives little opportunity for learning, whereas the three higher levels provide ample opportunities for learning.

In order for an organization with long teaching traditions to be able to move up to the levels that really give a good gain realization and can give the NAF the modern education that is desired, it is required that management has realistic expectations of what a major restructuring will require of time and effort. It is not just about initiating the measures, they must also be followed up over time and support must be given to try out new behaviors to solve the teaching tasks adapted to a new regime. This is also a separate success criterion for a change process to succeed in general (Yukl and Lepsinger, 2004). When dwelling upon the goals set for leaders shown in Table 7 again they might seem a bit overambitious and perhaps not easy to obtain. In general, it requires strategic planning and systematic follow-up, as well as practical facilitation for management, to fulfill these goals. It is largely about activating the teachers so that they get a designated direction and experience a decent amount of support that gives them the willingness to cope with challenges. A supportive academic environment must be created where successes are recognized and unsuccessful attempts are accepted for learning from experience.

## Materials and Methods

### Data Collection

We collected data that we thought could answer our three hypotheses. We used a document analysis to answer our first hypothesis. To answer our second hypothesis we used oral and written data collected from key personnel that were involved in the digitization process within the NDUC. In order to investigate our third hypothesis, we investigated answers given by the participants (cadets) that were enrolled in the Basic Officer Education. The participants had been taking part in the different courses that were common in the new Basic Officer Education at the three military academies in Norway. This data collection took place after each of the three courses that our participants had taken part in at the Norwegian Military Academy (NMA) (Army). The three courses were joint courses for cadets from the Navy, Army, and Air Force, but the education was given at the premises of the NMA for all three branches. The purpose of investigating the answers that the participants gave to the questions about digitization in their education, was to examine whether the goals stated by the Norwegian Ministry of Education and Research (Kunnskapsdepartementet, 2017a) regarding digitization, learning and teaching could be traced throughout the levels of the military educational system, or if they became lost in transition along the line.

The study was limited to looking at leadership, digitization, processes and organization and not intended as an interpretation of trends of the new Basic Officer Education. The research we conducted may potentially reveal conditions that the organization may be criticized for. Criticizing a system that you are a part of can be perceived as problematic for some. In order to get the most honest answers, our participants were therefore anonymous when filling out the course evaluations. As such, our research approach can be described as a mixed methods approach (Creswell, 2014). Our approach consisted of a qualitative document analysis which also included quantitative data collected from the different documents. We also collected qualitative data in the form of oral and written information from key personnel involved in the digitization process at the NDUC. Finally, we used a quantitative approach as we collected data through three course evaluations from our participants.

### Document Analysis

In order to find out to what extent digitization is described in the government documents, the documents issued in the NAF, and the documents used in the NAF Basic Officer Education, we searched for the specific words digitization and digital tools in each document. Then we counted the number of times the words digitization and digital tools were mentioned in each document. The idea behind this approach was to see if any of the original demands regarding digitization and the use of digital tools disappeared in the dissemination of the documents from the Ministry to the NAF Basic Officer Education.

The most important documents from the Norwegian government used in the document analysis have been the report “Quality Culture in Higher Education” (Kunnskapsdepartementet, 2016), the Kunnskapsdepartementet (2017a) report “Digitization Strategy for the University and Higher Education 2017–2021,” “the ICT strategy for education” (Kunnskapsdepartementet, 2017c), and “the Concentration for quality – Structural reform in the university and college sector” (Kunnskapsdepartementet, 2014).

From the NAF we used several documents for document analysis. The most important ones issued from the NAF’s highest governing level were the “Proposition to the Norwegian government (proposal for parliamentary resolution) Campaign and sustainability Long-term plan for the defense sector,” issued by the Ministry of Defence (Forsvarsdepartementet, 2016a), the Norwegian government report 14 “Competence for a new era” (Forsvarsdepartementet, 2013), and the report the “Norwegian Armed Forces digitization strategy” (Forsvarsstaben, 2018).

From the NDUC level, we used the NMA’s digitization strategy (Krigsskolen, 2014). From the NDUC, we also used the course descriptions and learning outcome descriptions (LODs for the three different joint courses in the Basic Officer Education; Norwegian Defence University College, 2018a,b,c).

**FIGURE 3:**
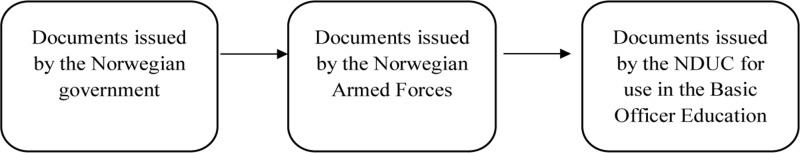
A conceptual model of the trace process regarding digitization.

Figure 3 reveals the conceptual model we used in order to trace the original demands regarding digitization from the Norwegian government down to the Basic Officer Education.

As far as we have been able to determine, the NAF did not issue other governing documents that took into account digitization during the time the present study was conducted.

### Data Retrieved From Key Personnel Within the NDUC

As part of the method of identifying the organization’s strategy documents on digitization, we requested to speak with relevant key personnel. These functioned as a quality manager at the Norwegian Military Academy, as head of the NDUC’s Advanced Distributed Learning (ADL) section, and as the officer responsible for the NMA’s digitization strategy. The inquiries were communicated both in the form of informal conversations and via e-mail.

### Answer to Questions From the Course Evaluations

Data was collected from the participants that had taken part in three joint courses in the new Basic Officer Education in the period from August 2018 until December 2018. These three joint courses had participants from the Norwegian Army, Air Force, and Navy. As part of the quality control of the new education, the participants were thus required to fill out an evaluation form after having completed each course.

### Participants

We choose to describe the persons that answered the questions in our study as participants, in line with Morse’s (1991) thinking that the term participants symbolize a more active engagement from the persons being studied, and that the term is commonly used in qualitative research.

Data was collected from three course evaluations that the cadets were requested to answer directly after having completed each of the three joint courses in their Basic Officer Education. 149 participants answered the questionnaire after completing the course “*The officer, state and society*”. Hundred and thirty two participants answered the questionnaire after having completed the course “*The officer and war*,” whereas 109 participants answered the questionnaire after finishing the course “*The officer as a lea*der.” The total number of cadets is classified and their total number, age and gender will therefore not be revealed here due to security concerns.

### Questions About Digitization Asked in the Course Evaluations

After each of the three courses, all cadets had to fill out an evaluation form. The evaluation form consisted of a total of 17 questions, and six of these questions were related to the digitization taking place in the teaching at the Basic Officer Education. The five first questions related to digitization required a quantitative answer, coded as Yes, No, or Do not know. The sixth question about digital learning resources was: “How have you used the digital learning resources?”. Participants were here requested to give a free text answer. However, we chose not to include this question in our analyses as we were not interested in how the digital tools were used by our participants, but only if they had been used in teaching. The five first questions related to digitization would provide adequate answers to this.

The Norwegian word *samhandling* was used in question 4, and it has no specific equivalent meaning in English, although it is closely connected to and resembles well the concept interaction in English. We therefore chose to use the concept interaction instead of the concept samhandling further in this article, in order to facilitate the understanding of our readers. Table 10 reveals the five questions related to digitization of teaching that was asked in each of the three course evaluations.

**TABLE 10:** Questions that were asked about the digitization of teaching in the three joint courses (authors’ translation).

(1) Has the course used the following digital learning methods been used in teaching? – Video lectures^1^ (2) Has the course used the following digital learning methods in teaching? – Simulations^1^ (3) Has the course used the following digital learning methods in teaching? – E-learning courses^1^ (4) Has the course used the following digital learning methods in teaching? – Online interaction on the Internet^1^ (5) Has the course used the following digital learning methods in teaching? – Video meetings^1^

*^1^Answer categories were either Yes, No, or I do not know.*

As can be seen from Table 10 the same question “Has the following digital learning methods been used in teaching?” was asked five times. For these five questions, however, there was a reference to a specific digital tool mentioned for each of the questions, which is respectively, video lectures, simulations, E-learning courses, Online interaction on the Internet, and video meetings. The participants could either answer Yes, No, or Do not know for each of these five questions.

### Procedure

First, we studied the governing documents issued from the Norwegian government regarding the digitization processes that was demanded. A second step was to study the governing documents for the NAF sector. Our third step was to study the governing documents that were specifically relevant for the NAF Basic Officer Education. Our fourth step was to collect information from key personnel at the NDUC that were involved in implementing the digitization process. The last step was to look at the answers to three course evaluations given by the participants that had taken part in the new education. We selected to study three specific courses, as these three courses were joint courses for cadets from the NMA, the Royal Norwegian Air Force Academy, and the Royal Norwegian Naval Academy.

### Ethical Considerations

A permission was granted to conduct the study from senior commanders at the NMA. The key personnel at the NDUC that were involved in implementing the digitization process and that gave us data was not anonymous. The study and research administration at the NDUC also gave their approval for us to use the course evaluations for the purposes of this study (E. Tveten Engan, personal communication, 05 February 2019). Approval was thus given to us from the people responsible for the data. Written informed consent was obtained from the participants that took part in the three courses, as they had given their informed consent earlier to fill out the course evaluations when they started their officer education, consenting to the course evaluations being a part of the quality assurance system at the NDUC and consenting to the data from the course evaluations being available for statistical and research purposes. The course evaluations were thus part of the current ongoing quality assurance system for the Basic Officer Education. Filling out the course evaluation was voluntary and the participants were informed that they could withdraw from filling out the course evaluations at any time, that no further questions would be asked and that they were completely anonymous.

The following general information was given to the participants before filling out the respective course evaluations on the digital platform Its learning: “As part of the quality assurance system at the Norwegian Defence University College, we want you to answer some questions about various aspects of the teaching of the topic. The results of this study provide the teachers and the college with a basis for future planning and further development of the course and study program. This investigation will form the basis for course evaluation in this topic. The survey is anonymous, i.e., your identity cannot be linked to your answers. The survey is based on a general questionnaire from the Norwegian Defence University College”.

We approached the Regional Ethical Committees for Medical and Health Research Ethics (REC) in Norway. As our study was not concerned with medical and health research projects, general and thematic research biobanks, and as we did not need dispensation from professional secrecy requirements for other types of research, the conclusion from REC was that we did not need to apply to an ethical committee in order to conduct our research (REC, personal communication, 04 January 2019, authors’ translation).

We also contacted the Norwegian Social Science Data Service (NSD). A part of the NSD is the Data Protection Services, and we submitted an online application to the NSD Data Protection Services to check whether approval to conduct the study was needed. In accordance with the NSD regulations, we specifically filled out the online application named *Notification form for treatment of personal information* to check if we needed to apply to an ethical committee to conduct our research. A total of twelve questions had to be answered in this notification form, and on all 12 we answered no, indicating that we did not plan to collect any personal data. Based upon our twelve answers posed by the NSD online application, the NSD Data Protection Services deemed that it was not necessary to fill out a regular application form in order to conduct our study. We received the following answer from the NSD: *You have stated that no personal data will be processed in the project. If you only want to process anonymous information, you do not need to report the project. An anonymous data material consists of information that cannot in any way identify individuals, either directly, indirectly or via e-mail/IP address or by code key* (NSD, personal communication, 08 January 2019, authors’ translation). The study thus did not need to be approved by the NSD, as we did not collect any personal data. An ethics approval was not required as per applicable institutional and national guidelines and regulations.

### Validity

According to Lincoln and Guba (1985), whether our collected data, analyses and findings can be transferred or generalized comes down to whether they can be used in similar and appropriate situations. The Basic Officer Education is a comparatively small part of the NAF, and therefore, we cannot generalize our results. On the other hand, our results give a view of the current status within the NAF regarding the perceived level of digitization and leadership challenges.

### Data Analysis

IBM SPSS 25.0 was used in order to investigate the answers that our participants gave to the questions related to digitization in the three course evaluations for the new Basic Officer Education. One-way analyses of variance (ANOVAs) were conducted for the answers that the participants gave to the five questions in the different course evaluations. Additional *post hoc* analyses (Tukey HSD) were also performed in order to investigate the differences between the course evaluations. Effect sizes (η^2^ = Eta squared) was also calculated.

## Results

The following sections will first describe the results from the document analyses related to the disappearance of original demands regarding digitization through the different levels of governing documents. Our search method was based upon searching for and counting the number of times the specific words digitization and digital tools was mentioned in the text in each of the different documents. Then, we describe the answers we received related to the lack of a plan to implement new digital tools in the military officer education, and finally the answers from our participants to the five questions related to use of digital tools in the teaching in the course evaluations. The descriptions of these three results sections thus correspond to our three hypotheses stated earlier. In short, our main finding was that there exists a loss of several of the requirements for digitization in the documents from the Ministry to the NAF Basic Officer Education. This may create some challenges when it comes to leading in the military educational organization.

### Hypothesis 1: Loss of Original Demands Regarding Digitization Through the Different Levels of Governing Documents

Our first hypothesis was: Several of the requirements for digitization have disappeared in the dissemination of the documents from the Ministry to the NAF Basic Officer Education.

### Documents Issued From the Norwegian Government

#### Quality Culture in Higher Education

The report “Quality Culture in Higher Education” (Kunnskapsdepartementet, 2016) refers to there still being, both nationally and internationally, a lack of research on what is needed for the students to achieve defined learning outcomes and personal development. The education must be experienced as relevant and prepare the students for their future occupation and must be carried out as effectively as possible. This report was issued before the Norwegian Ministry of Education and Research’s (Kunnskapsdepartementet, 2017a) report “Digitization Strategy for the University and Higher Education 2017–2021” that was published in 2017. The word digitization was in this document mentioned 10 times in total. The analysis of this document also revealed that the phrase digital tools was mentioned only five times in the document.

#### Digitization Strategy for the University and Higher Education 2017–2021

The Norwegian Ministry of Education and Research’s (Kunnskapsdepartementet, 2017a) report “Digitization Strategy for the University and Higher Education 2017–2021,” shows that digitization in higher education is an important focus area for the government in order to create the basis for society’s further development of expertise. This must originate from the education sector so that practitioners have acquired sufficient competence when they enter their future work life. The strategy defines goals for several levels in the universities and concludes that the organizations must prepare their own strategy plans for digitization, and the work must be anchored in the management. It is especially important to ensure that the staff has the necessary expertise. The word digitization was mentioned 116 times in the document, whereas the phrase digital tools was mentioned three times in the document.

#### ICT-Strategy for Education

The word digitization was mentioned 48 times and the phrase digital tools was mentioned 7 times in the document “*ICT-strategy for education*” (Kunnskapsdepartementet, 2017c).

#### Concentration for Quality – Structural Reform in the University and College Sector

The word digitization was mentioned five times in the document “*Concentration for quality - Structural reform in the university and college sector*” (Kunnskapsdepartementet, 2014). The phrase digital tools was not mentioned, but one reference to electronic tools was found.

#### Campaign and Sustainability Long-Term Plan for the Defense Sector

In the “Proposition to the Norwegian government (proposal for parliamentary resolution) Campaign and sustainability Long-term plan for the defense sector,” issued by the Ministry of Defence (Forsvarsdepartementet, 2016a), the word digitization was mentioned five times. The phrase digital tools was not mentioned, although we found a peripheral reference to the need for development of methods and tools related to digital ICT security concerns.

### Documents Issued From the NAF

#### The Norwegian Government Report 14 “Competence for a New Era”

The Norwegian government report 14 “Competence for a new era” (Forsvarsdepartementet, 2013) states that “the defense sector is facing a new phase in a long transition process. It is about the sector’s most important resource, the people. It is about putting expertise in the center” (p. 7, authors translation). The word digitization and the phrase digital tools were not mentioned in this report.

#### The NAF Digitization Strategy

The NAF digitization strategy (Forsvarsstaben, 2018) is a report issued by the Norwegian Defence Staff. It deals with the NAF’s overall need to digitize to be up-to-date and able to fulfill the mandate they are given by society. It signals five focus areas and aims to provide increased operational capability and internal efficiency. Digital competence is one of five priority areas and the area we have focused on here. The word digitization was mentioned 103 times in this document, and the phrase digital tools was mentioned five times.

#### The Norwegian Military Academy’s Digitization Strategy

The NMA’s digitization strategy (Krigsskolen, 2014) describes the NMA’s meeting with the “digital natives” who have grown up with the Internet, and what is needed to ensure these cadets get relevant and interesting learning. It also addresses the challenges this entails for teachers and management. The strategy document reveals that the word digitization and the phrase digital tools were not mentioned at all in the document.

#### Course Descriptions for the Basic Officer Education

Looking at the three course descriptions for the joint courses *The officer, state and society*, *The officer and war*, and *The officer as a leader*, we found very little that could be traced back to the digitization strategy issued from the Ministry of Education and Research (Kunnskapsdepartementet, 2017a). The word digitization or the use of digital tools were not mentioned in the course description for the course *The officer, state and society*. Neither were any of the learning objective descriptions (LODs) related to digitization or the use of digital tools (Norwegian Defence University College, 2018a). Digitization was only mentioned once in the course description for the course *The officer and war* where it is stated that the students must also expect some individual digitized work tasks. None of the LODs was related to digitization or the use of digital tools (Norwegian Defence University College, 2018b). Regarding the course *The officer as a leader*, digitization or the use of digital tools were not mentioned in the course description or in any of the LODs (Norwegian Defence University College, 2018c). The phrase digital tools was also not mentioned at all in any of the three course descriptions. However, there was a reference to the use of individualized digitized work tasks in the course description for the course *The officer and war*. We thus chose to interpret this statement as a demand that forced the participants to use digital tools, counting this as a reference to the use of digital tools.

Table 11 reveals the number of times the words digitization and digital tools were mentioned in the different documents examined in the study.

**TABLE 11:** Number of times the words digitization and digital tools were referred to in documents from the government, the NAF, and the NDUC.

**Documents issued by the government**	**Digitization**	**Digital tools**
1. Quality culture in higher education	10	5
2. Digitization strategy for the University and Higher Education 2017-2021	116	3
3. ICT-strategy for education	47	7
4. Concentration for quality - Structural reform in the university and college sector	0	0
5. Campaign and sustainability Long-term plan for the defense sector	5	0
**Documents issued by the NAF**		
6. The Norwegian government report 14 “Competence for a new era”	0	0
7. The NAF digitization strategy	103	5
**Documents issued by the NDUC**		
8. The NMA’s digitization strategy	0	0
**Course descriptions for the Basic Officer Education**		
9. Officer, state and society	0	0
10. Officer and war	0	1
11. Officer as a leader	0	0

Table 11 clearly reveals that there existed a loss of the original demands regarding digitization. This can clearly be seen as the number of references to digitization and digital tools drops as one goes down in the document hierarchy from the Norwegian government to the NAF and then to the NDUC and documents for use in the new Basic Officer Education. This as the number of references to either digitization or digital tools simply disappear from document to document from the Ministry down to the Basic Officer Education.

Our first hypothesis received support as our document analyses revealed that were not many of the original goals stated in the digitization strategy that could be retrieved down at any of the LODs in the documents issued for the Basic Officer Education.

### Hypothesis 2: Lack of a Plan to Implement New Digital Tools in the Basic Officer Education

Our second hypothesis was*: No plan has been prepared as part of the educational change process for the introduction and implementation of new digital tools in the NAF Basic Officer Education.*

We sent e-mail to key personnel involved in the digitization processes in the NDUC. Data was collected through the means of both informal conversations and via e-mail.

The quality manager at the NMA knows the overall documents and is concerned with the theme, and believes no overall strategy for digitization has yet been prepared, but that the topic is known and acknowledged (B. K. Haugdal, personal communication, 20 December 2018).

The head of the NDUC’s Advanced Distributed Learning (ADL) section said that an overarching digitization strategy for the college is under preparation right now (G. Isaksen., personal communication, 04 February 2019).

The preparation of the new strategy plan for digitization in education in the NDUC takes into account both the report on digitization from the government and the quality report. It also benchmarks what other universities and colleges have done. Currently, there are different perceptions of what digitization entails. The responsible officer stated that digitization will be a great renewal and transformation that will affect many areas. He also commented on the importance of anchoring in the management in order for implementation to succeed (A. Broberg, personal communication 05 February 2019).

The officer responsible for the NMA’s digitization strategy stated that the NMA had prepared a strategy for digitization that was put aside when the transition process started. It became clear that the school was to be placed organizationally under the NDUC, and it was decided that the further digitization work should be coordinated (R. Wold., personal communication, 02 February 2019).

Our second hypothesis also received support as it became clear that no plan had been prepared as part of the educational change process when it came to introducing and implementing new digital tools in the Basic Officer Education.

### Hypothesis 3: Lack of a Systematic Introduction and Implementation of Digital Tools in the Education

Our third hypothesis was*: It is up to the individuals to introduce and implement the use of new digital tools in the NAF Basic Officer Education.*

In order to find out whether the use of digital tools was implemented in the Basic Officer Education, we collected data from the participants of the three joint courses in the Basic Officer Education. The participants were asked a total of five questions that dealt with digitization in relation to teaching. Below we will reveal the results from these five questions for each of the three course evaluations.

#### Digitization of Teaching

Tables 12–14 reveals the number of Yes, No, and Do not know answers, and percentages of these answers to the five questions related to digitization of teaching that were asked in each of the three course evaluations, respectively the course *The officer, state and society* (Table 12), *The officer and war* (Table 13), and *The officer as leader* (Table 14).

**TABLE 12:** Answers to five questions that were asked about the digitization of teaching in the course *The officer, state and society*.

**Question: Have the following digital learning methods been used in teaching?**						

	**Yes**	**%**	**No**	**%**	**Do not know**	**%**
1. Video lectures^1^	90	60.4	55	36.9	4	2.7
2. Simulations^2^	4	2.7	122	81.9	22	14.8
3. E-learning courses^2^	6	4.0	123	82.6	19	12.8
4. Online interaction on the internet	59	39.6	66	44.3	24	16.1
5. Video meetings^2^	0	0.0	146	98.0	2	1.3

*Scale ranging from 1 (Yes) to 2 (No), with an alternative answer 3 (Do not know). ^1^*n* = 149, ^2^*n* = 148, percentages estimated from *n* = 149, missing 1 (0.7%).*

**TABLE 13:** Answers to five questions that were asked about the digitization of teaching in the course *The officer and war* (*n* = 132).

**Question: Have the following digital learning methods been used in teaching?**						

	**Yes**	**%**	**No**	**%**	**Do not know**	**%**
1. Video lectures	71	53.8	52	39.4	9	6.8
2. Simulations	32	24.2	84	63.6	16	12.1
3. E-learning courses	5	3.8	115	87.1	12	9.1
4. Online interaction on the internet the Internet	46	34.8	60	45.5	26	19.7
5. Video meetings	3	2.3	122	92.4	7	5.3

*Scale ranging from 1 (Yes) to 2 (No), with an alternative answer 3 (Do not know).*

**TABLE 14:** Answers to five questions that were asked about the digitization of teaching in the course *The officer as a leader*.

**Question: Have the following digital learning methods been used in teaching?**						

	**Yes**	**%**	**No**	**%**	**Do not know**	**%**
1. Video lectures^1^	90	82.5	16	14.7	3	2.8
2. Simulations^1^	16	14.7	66	60.5	27	24.8
3. E-learning courses^2^	13	11.9	72	66.1	22	20.2
4. Online interaction on the Internet^3^	39	35.8	52	47.7	17	15.6
5. Video meetings^3^	2	1.8	98	89.9	8	7.3

*Scale ranging from 1 (Yes) to 2 (No), with an alternative answer 3 (Do not know). ^1^*n* = 109, ^2^*n* = 107, percentages estimated from *n* = 109, missing 2 (1.8%), ^3^*n* = 108, percentages estimated from *n* = 109, missing 1 (0.9%).*

#### Evaluations From the Course the Officer, State and Society

Table 12 reveals the answers to five questions that were asked about the digitization of teaching in the course *The officer, state and society*.

As can be seen from Table 12 the answers to the five questions about the digitization of teaching reveals some interesting findings. Over 60% of the participants (60.4%) answered that video lectures were used in teaching during the course, whereas 2.7% answered that simulations had been used in teaching during the course. Furthermore, 4.0% of the participants answered that E-learning courses were used in teaching during the course. Regarding the question if online interaction on the Internet was used in teaching during the course 39.6% of the participants answered yes to this question. None of the participants said that video meetings were used in teaching during the course. Taking a closer look at the percentages of participants that stated Do not know to the five questions, this ranges from 1.3% to 16.1%. This can be interpreted as an indication that our participants did not understand or comprehend the meaning of the different digital tools that were mentioned in the five questions. It may also be that they do not understand why the question was asked when the course description does not include the introduction of such digital tools.

#### Evaluations From the Course the Officer and War

Table 13 reveals the answers to five questions that were asked about the digitization of teaching in the course *The officer and war*.

As can be seen from Table 13 more than half of the participants (53.8%) answered that video lectures were used in teaching during the course. Regarding the use of simulations, only 24.3% of the participants stated that this had been used in teaching during the course. Only 3.8% of our participants said that E-learning courses were used in teaching during the course. However, regarding, 34.8% of the participants answered that online interaction on the Internet was used in teaching during the course. Finally, only 2.3% of the participants said that video meetings were used in teaching during the course. Looking at the percentages of participants that stated Do not know to the five questions, this ranges from 5.3% to 19.7%. This may be an indication that our participants simply did not know the meaning of the different digital tools asked about in the five questions.

#### Evaluations From the Course the Officer as a Leader

Table 14 reveals the answers to five questions that were asked about the digitization of teaching in the course *The officer as a leader*.

Table 14 reveals that 82.5% of the participants answered that video lectures had been used in the teaching during the course. Only 14.7% said the same regarding the use of simulations, and 12.1% said that E-learning courses were used in the teaching. Regarding online interaction on the Internet, 36.1% of the participants answered that this was used in the teaching during the course. Looking at the use of video meetings, only 1.9% answered that this had been used during the teaching. Taking a closer look at the percentages of participants that stated Do not know to the five questions, this goes from 2.8% to 24.8%. Again, the high percentage of participants that answered that they did not know whether simulation, e-learning courses and online interaction on the Internet may again indicate that the participants did not fully comprehend the meaning of the different digital tools that they were asked to consider in the five questions. Alternatively did not understand why the question was asked when the course description did not include the introduction of such digital tools.

One-way analyses of variance (ANOVAs) were conducted for the answers given to the five questions in the three different course evaluations. A significant difference was found between the answers given to the three course evaluations for the question Has the course used the following digital learning methods in teaching? Video lectures, as determined by a one-way ANOVA, *F*(2,389) = 10.74, *p* < 0.001, MS_*e*_ = 3.29, η^2^ = 0.053. Statistical power to detect an effect for the differences in the use of video lectures between the three courses with an alpha of 0.05 was at 0.99. As such, it is above the minimum acceptable level at 0.80 as stated by Cohen (1988). In addition, a significant difference between the three courses was found for the question Has the course used the following digital learning methods in teaching? Simulations, indicating differences in the use of simulations between the courses, *F*(2, 388) = 8.30, *p* < 0.001, MS_*e*_ = 2.40, η^2^ = 0.041. Statistical power to detect an effect for the differences in use of simulations between the three courses, with an alpha of 0.05 was at 0.96, and again above the minimum acceptable level at 0.80. For the remaining three questions, no significant differences were yielded. The question Has the course used the following digital learning methods in teaching? E-learning courses yielded no differences in use in the three courses, *F*(2,386) = 0.25, *p* = 0.78, MS_*e*_ = 0.05, η^2^ = 0.001. Statistical power to detect an effect for the differences in use of E-learning courses between the three courses, with an alpha of 0.05 was at 0.08. As such, substantially below the minimum acceptable level at 0.80. Regarding the question Has the course used the following digital learning methods in teaching? Online interaction on the Internet, no significant differences between the courses were found: *F*(2, 238) = 0.49, *p* = 0.62, MS_*e*_ = 0.162, η^2^ = 0.003. Here, the statistical power to detect an effect for the differences in use of online interaction on the Internet between the three courses, with an alpha of 0.05 was at 0.13, and also well below the minimum acceptable level at 0.80. Finally, the question Has the course used the following digital learning methods in teaching? Video meetings, also revealed no significant effects, *F*(2,387) = 0.99, *p* = 0.537, MS_*e*_ = 0.06, η^2^ = 0.005. Also, for this question, the statistical power to detect an effect for the differences in use of video meetings between the three courses, with an alpha of 0.05 was at 0.05, and again very low. Cohen (1988) has provided benchmarks to define small (η^2^ = 0.01), medium (η^2^ = 0.06), and large (η^2^ = 0.14) effect sizes. We found small effect sizes for the question related to video lectures and for the question related to simulations, and even smaller effect sizes for the remaining three questions related to E-learning courses, online interaction on the Internet, and video meetings.

Table 15 below sums up the percentages of participants that answered Yes to the five questions about the use of digital learning methods in the teaching in the three course evaluations.

**TABLE 15:** Percentages of answers with Yes to the five questions that were asked about the use of digital learning methods in the teaching in the three course evaluations.

**Question: Have the following digital learning methods been used in teaching?**			

**Course evaluations**	**The officer, state and society %**	**The officer and war %**	**The officer as a leader %**
1. Video lectures	60.4	53.8	82.5
2. Simulations	2.7	24.2	14.7
3. E-learning courses	4.0	3.8	11.9
4. Online interaction on the internet	39.6	34.8	35.8
5. Video meetings	0.0	2.3	1.8

Furthermore, *post hoc* analyses (Tukey HSD) were performed to investigate the differences between two and two course evaluations. Analyses were thus conducted for the answers given to the five questions on the use of digital learning methods (see Table 16).

**TABLE 16:** Results of the conducted *post hoc* analyses (Tukey HSD) for the five questions Have the following digital learning methods been used in teaching? in the three course evaluations.

**Course evaluation**	**Course evaluation**	**MD^1^**	**Sig.^2^**
**Video lectures**			
The officer, state and society	The officer and war	–0.11	0.24
	The officer as a leader	0.22^∗^	0.01
The officer and war	The officer as a leader	0.33^∗^	0.00
**Simulations**			
The officer, state and society	The officer and war	0.24^∗^	0.01
	The officer as a leader	0.02	0.95
The officer and war	The officer as a leader	−0.22^∗^	0.01
**E-learning courses**			
The officer, state and society	The officer and war	0.04	0.78
	The officer as a leader	0.00	1.00
The officer and war	The officer as a leader	–0.03	0.85
**Online interaction on the internet**			
The officer, state and society	The officer and war	–0.08	0.59
	The officer as a leader	–0.03	0.94
The officer and war	The officer as a leader	0.05	0.84
**Video meetings on the internet**			
The officer, state and society	The officer and war	–0.02	0.82
	The officer as a leader	–0.04	0.34
The officer and war	The officer as a leader	–0.03	0.69

*^1^MD, mean differences. ^2^Sig., significance level. ^∗^*Post hoc* analyses (Tukey HSD) indicated significant differences at *p* < 0.05 level.*

As can be seen from Table 16, the use of video lectures revealed that there were no significant differences of percentages of use between the course *The officer, state, and society* (60.4%) as compared to the course *The officer and war* (53.4%). A significant result was that we found differences in percentages of use regarding the use of video lectures between the course *The officer, state and society* (60.4%) and the course *The officer as a leader* (82.5%). In addition, regarding the use of video lectures in teaching, we found a significant difference in percentages between the course *The officer and war* (53.4%) and the course *The officer as a leader* (82.5%). Furthermore, the use of simulations in teaching also yielded a significant difference of percentages of use between the course *The officer, state and society* (2.7%) and *The course officer and war* (24.2%) and between *The course officer and war* (24.2.%) and the course *The officer as a leader* (14.7%). No significant difference in percentages in use of simulations in teaching was revealed between the course *The officer, state and society* (2.7%) and the course the officer as a leader (14.7%). Regarding the three other questions, that is, the use of E-learning courses, Online interaction on the Internet and Video meetings on the Internet, no significant differences in percentages of use of these digital learning methods were found between the three different courses.

It is important to note that a significance test does not tell the size of a difference between two measures (that is, its practical significance), nor can it easily be compared across studies. Consequently, caution must be taken here when interpreting our results, as the effect sizes measured as η^2^ (Eta squared) were small, thus indicating relatively small practical differences.

## Discussion

A common finding in the research literature is that if one wants to spread expertise, the most effective way of doing this is through systematic dissemination. This happens by creating an environment for sharing knowledge (Chyi Lee and Yang, 2000). Whether this has been taking place in the process of implementing digitization in the NAF Basic Officer Education, was the main question we sought to find the answers to.

### Hypothesis 1: Loss of Original Demands Regarding Digitization Through the Different Levels of Governing Documents

Our first hypothesis: Several of the requirements for digitization have disappeared in the dissemination of the documents from the Ministry to the NAF Basic Officer Education gained support.

The article has focused on one of the four main goals of the national digitization strategy; high quality in education and research. The current state is that the education mainly takes place quite traditionally at the NDUC. There are fewer teachers and the student mass has increased in parts of the education. This has made it more demanding to attend to the desired quality of education in some parts of the study where, among other things, the cadet’s personal development is in focus. As previously shown in Table 2, the Norwegian government defines quality in higher education by describing the ambitions of the students’ learning (see Table 17).

**TABLE 17:** Ambitions of the students’ learning as stated in the Norwegian government’s definition of quality in higher education (Kunnskapsdepartementet, 2014–2015) (authors’ translation).

Students should: (1) Achieve the best possible learning outcomes and personal development (2) Encounter relevant education that prepares them well for active participation in a diverse and democratic society and for a future professional career (3) Achieve the education as effectively as possible.

In the NAF, the studies are organized in a way that makes it possible to ensure that the cadets graduate in the stipulated time. The studies are designed to be close to practice and emphasis is placed on both reflection and critical thinking in the performance. In this study, we have not taken into account the feedback the school has received from receiving units on newly qualified cadets’ expertise. Generally, it is said that they are well prepared for the service and emphasis is placed on the fact that they will continue to learn after the end of their studies. These ambitions gathered under the heading: learning outcomes, relevance and implementation are well taken care of within the existing tradition.

When it comes to the five characteristics of quality of education, that is, high ambitions on behalf of the students, engaging and varied learning activities, quality culture and clear education management, integration of students in the academic community and interaction with working life, the NAF has high ambitions on behalf of the students. Varied learning methods are used and the teachers focus on quality in their teaching preparations. It is emphasized that the teaching should as far as possible be research-based, but there is little time allocated to own research and development. Since the NDUC educates professional practitioners to their own organization, there is naturally good contact between studies and the coming work life. In recent years, the school has also emphasized providing all teaching staff with basic pedagogical competence. It therefore appears that it is only when the NAF comes face to face with the government’s digitization strategy that we cannot find the expected efforts in the organization.

As the goals stated in the digitization strategy from the Ministry of Education and Research seemed to have been lost in transition down to the documents used in the NAF, it is possible that the available competence and resources found in the military educational sector simply is not synchronized with the strategy. It is also natural to assume that the reason why digitization in education has not yet been emphasized systematically and anchored in management is due to the large educational reform. There was a lot that was changed and many people were affected. Personnel who could no longer find their jobs in the new organization were helped to find new positions and very few were left without work. New educational programs were to be prepared in a very short time and therefore therefore, there was not time to think about how to train the staff to apply and understand digital aids in the teaching, and far less transform their teaching practice. Solving this situation should therefore be a strategic measure given the institutions’ academic freedom and autonomy. As quoted in the Ministry of Education and Research strategy on digitization of higher education.

The new and complex information landscape with extensive use of data and technology involves extensive challenges of an ethical, legal and security nature. It places increased demands on ICT competence, accountability, digital judgment and the ability to source criticism at all levels. In line with the main principles of the government’s digitization policy, it is the users and their needs that should be the central starting point (Kunnskapsdepartementet, 2017a, p. 5, authors translation).

However, the road map of how to get from the Ministry of Education and Research strategy on digitization of higher education to the NAF is missing important parts. As such, the mismatch between the organization’s requirements and needs and the competence the employees possess resembles what Lai (2004) refers to as *incongruence competence*, or incompetence. This is also in line with the NAF’s own digitization strategy, which states that the organization’s level of maturity regarding digitization is currently low. A local digitization strategy has not yet been prepared for the NDUC. Training is given on request sporadically. There are skills in a few and there are some enthusiasts who try out solutions they know. The ADL office is willing to assist and share their expertise on request but they are quite few. When the college is to proceed with the development and implementation work required by the Ministry of Education and Research (Kunnskapsdepartementet, 2017a), this must probably be executed in collaboration with the teaching staff.

### Hypothesis 2: Lack of a Plan to Implement New Digital Tools in the Basic Officer Education

Our second hypothesis also received support. We discovered that no common strategic plan had been prepared for the digitization of the education in the NAF.

In 2014, the management of the NMA prepared a strategy for the use of technology in the education. However, this was set aside when the colleges were merged and the NDUC wanted everyone to use their digitization strategy. A new strategy document was not prepared at that time. For the NMA, this meant that a recently started development process got a relatively abrupt stop. Dokter and Aspelund (2017) emphasize that the leadership at the NMA also has clarified their commitment to the digitization strategy from the Ministry of Education and Research (Kunnskapsdepartementet, 2017a), by issuing a Standing Order (#005) regarding the use of information technology. The Standing Order specifically mentions OneNote and despite security concerns states that the NMA will use OneNote Class Notebook as a tool for pedagogical interaction and learning. The Standing Order does not discuss how OneNote is to be used to achieve this objective and the use of OneNote is limited, in this case, to non-formal communication. However, conflicting information is provided from the NDUC centrally which points out that It’s learning should be used instead of OneNote. Information about the use of this learning platform is given orally to those who are responsible for planning the teaching in the new education. Information on where to get support and by whom is also given orally. There is still a lot that can be improved in evaluating the various topics in the new education. However, we can conclude that there must have been an expectation from the NDUC that it should be adapted for digitization in the teaching since five questions specifically ask which digital tools have been used, such as video lectures, and interaction on the Internet. In addition, it is asked how the cadets have used the same tools. We have omitted these answers in this study since it is beyond our research questions.

### Hypothesis 3: It Is Up to the Individuals to Introduce and Implement the Use of New Digital Tools in the NAF Basic Officer Education

Finding answers to our third hypothesis proved to be more difficult. The third hypothesis stated: It is up to the individuals to introduce and implement the use of new digital tools in the NAF Basic Officer Education. When we studied the course plans and the communication between teacher and cadets, we could see that there were big differences in what the teachers had used of digital aids. When we then compared this result with our previous hypothesis that no plan for digitization existed, we concluded that it is still up to each enthusiast to use digital aids in the teaching. We therefore choose to conclude that hypothesis 3 received partial support.

### The Use of Digital Learning Resources in the Three Joint Courses

Some teachers seem to have used some digital tools as we mentioned above, thus implementing the proposed digitization strategies, whereas other teachers seem to have done this to a lesser degree. It also seems that the participants does seem to suffer from a lack of understanding as to what digitization really means or they have used digital teaching methods that are not planned by the teacher. This result may indicate that it is up to the enthusiasts to digitize the learning methods still. We also see that it is video lessons that are mainly used so this supports NAF’s analysis that the organization still has low maturity regarding digitization (Forsvarsstaben, 2018). The teaching continues largely as before, only supported by some digital aids.

As shown in Venkatraman’s (1994) model this illustrates that the teachers continue to do the same tasks only supported by technology. The work processes are only changed to a limited extent, for example, the syllabus may be digitized instead of distributed on paper and information may be provided via a learning platform instead of orally or by mail.

The figure “a new window for learning” (see Figure 2 in this article) by Elstad and Hafnor (2017) illustrates that the use of a learning platform provides little gain realization and requires relatively low degree of restructuring. Acquiring and sharing sufficient insight into how to use a learning platform has thus become a possible first step along the way. However, it is clear that the new NDUC is not yet synchronized with regard to which platforms to use and there are also different experiences at different campuses of which platform is the best and most flexible. This can also create some friction and uncertainty in the digitization implementation work. In order for the organization to be able to move toward complex learning systems this requires both time and competence to plan such an education program. The learning effect is to be increased through the use of several learning resources such as a rich and relevant selection of technology, other learning resources such as the practice community (co-workers at work) and fellow students (group work, correcting each other’s tasks).

### Motivation for Change and Development of Digitization

Technology will continue to play an important role in leadership and the development of soft skills in the military. As more military training and education programs find their way into virtual classrooms, self-paced e-learning courses, and webinars, many believe these new tools could change the way the military assess and develops its leaders in the future (Dobbs et al., 2017). Day et al. (2009) have discussed how advances in technology and alterations within contextual environments can be seen as a part of “continuous learning” and as an element of leadership development. Hopson et al. (2001) found that how the faculty used technology in laboratories and classrooms could improve students’ higher-order thinking skills. More specifically, critical thinking and problem-solving were the two areas that could be improved most with the use of technology. Student engagement, faculty presence, and overall cognition in a course were also enhanced if an appropriate combination of technologies was used (Garrison, 2007). For the military, technology enables the organizations to compress both time and space (Fun and Ang, 2017).

A strategy formulated by senior management and implemented by middle managers can thus encounter problems in the implementation. A general and ideal formulation from bureaucratic level will be perceived as inappropriate for the operational approach that seeks a detailed and solution-oriented practice (Johannessen and Glomseth, 2015). In other words, strategic management has different meanings depending on the practice.

Several master’s theses have been written on competence and strategic competence management in the NAF at both the University of Tromsø, Hedmark University College, Copenhagen Business School and the NDUC (Karlsen, 2007; Andreassen, 2008; Nilsen and Jørgensen, 2009; Sande, 2012; Pedersen and Gabrielsen, 2013; Flom, 2016; Furulund, 2016; Johansen, 2017). Common to the theses is that they largely point to the distance between the strategic management documents that are advocated by the NAF leadership and what is practiced in the organization. As mentioned before, even the NAF Cyber Defense has been found to struggle with issues such as strategic competence leadership, organizational structures, competence development, needs, plans, handling and communication (Boe and Torgersen, 2018).

The ability to digitally manage knowledge has become a must in order to support future soldiers with an adequate knowledge in military operations and trainings (Alkhred et al., 2018). Managing knowledge in a military environment thus includes the use of different digital tools, and the military educational sector should be at the forefront of using these tools. Through the development of information technology (IT), several digital systems have been created for knowledge creation, sharing and dissemination (Zainol et al., 2018). Examples of such digital systems are knowledge based systems (KBS), document management systems, relational databases, semantic networks, expert systems, decision support systems (DSS) and simulation systems (Dorasamy et al., 2013).

Leadership in the NAF is based largely on trust and cooperation. This has certain practical consequences in the daily interaction. There seems to exist an underlying belief in the NAF and the NDUC that if only orders, intentions, and visions have been verbalized orally, they will be executed. A leadership challenge connected to this is how this connects to structure and organization. The NDUC has emphasized close contact with the individual student in order to secure this person’s personal leadership development. After the major downsizing process recently, this opportunity will be reduced.

The change process has to some extent been experienced as top–down, with only the establishment of certain working groups that have handled sub-projects. This means that the employees have to a large extent been dependent on getting information via intranet or directly from the management. Cuts have been necessary to meet the demands of the reform. This may seem to cause the employees to strive to familiarize themselves with the new topics to be taught and choose to apply old practice. In addition, an assessment is possibly influenced by where in the system one is placed or what role one holds. “Where you stand depends on where you sit” (Allison, 1969, p. 711). This indicates that leaders at different positions in the NAF and the NDUC will see different challenges when it comes down to digitization of the educational sector in the NAF. It boils down to the leaders’ ability to influence and control the perceptions of employees’ motivation and create incentives to implement and use new digital tools. A hampering issue here could be the current skepticism and worries about cyber security within the NAF, resulting in leaders being slow to implement new technologies. Another unfortunate circumstance is the fact that the senior management of the NMA is replaced at the same time because of the natural turnover and bureaucratic recruitment and hiring routines.

### How to Implement and Lead the Digitization Process in the Military Educational Sector

Cram et al. (2015) point out that there seems to exist a limited understanding of the steps that different organizations need to take when implementing new information systems. In particular, they point to the challenges of avoiding negative performance implications such as employee resistance or process delays. In a report looking at the digital transformation at the new military education for officers in the NAF, Dokter and Aspelund (2017) point out that there are two basic facts that are important when reviewing recent research on digital transformation in the classroom. The first fact they point out is that digital transformation (digitization) will only succeed if management and leadership are committed to a strategy for digitization. The second fact they point out is that institutions need to place a high emphasis on fostering and building the digital competencies of their faculty staff. An internal analysis is needed of the staff’s ability and willingness to transform their teaching practices and thus achieve a measure of the organization’s maturity in terms of digitization. This is an important point, as it is well known that humans have a tendency to continue or maintain their previous actions (Samuelson and Zeckhauser, 1988). It is further also well known that the decisions one is about to make are anchored in previous decisions (Ritov and Baron, 1992). Kotter (1996) emphasizes the importance of creating a sense of urgency to gain the needed cooperation from the staff. However, the NMA has been relatively satisfied with the form and quality of the education since the last reform in 2012. If the staff does not feel the necessary urgency, the momentum of change may suffer. This may, for example, be due to the fact that the teaching staff have an internal and somewhat complacent focus and are not sufficiently oriented about what happens outside their own organization. Across the various campuses of the NDUC, this will vary, so it will also require a flexible approach to training and implementation.

Kotter (1996) further emphasizes the importance of establishing a guiding group that can be ambassadors and contribute to active implementation. In order for this group to have sufficient influence, it must have credibility among the employees, and sufficient power and expertise. It is necessary that the main line leaders participate. As a continuation of this line of thinking, Bergh (2017) has pointed out that it is important that one highlights the good experiences and examples in order to show others what is possible to achieve when it comes to digitization. In order to create the desired movement in the teaching staff, the management must have critical mass, that is, enough enthusiasts who are willing to learn and have a strong social position. These people can be the organization’s change agents. The goal must be to systematically increase this group of employees based on the enthusiasts’ efforts and willingness to share and help the others. At the same time, a steady management is needed, supplemented by sufficient time and competence building activities to bring experienced teachers with good pedagogical competence into the digital teaching space. Yukl and Lepsinger (2004, p. 112) give five guidelines for implementing change (see Table 18).

**TABLE 18:** Five guidelines for implementing change.

(1) Fill key positions with competent change agents (2) Prepare people to adjust to change and cope with the pain (3) Provide opportunities to celebrate early successes (4) Keep people informed about the progress of change (5) Demonstrate continued commitment to change

In practice, following this advice in the public sector can be complicated. It is desirable to have change agents in all important positions but it is legally and ethically challenging to move people out of their positions because of their resistance to a change process that may require a lot of them personally. Working life in Norway has sufficient rules to deal with the duty of loyalty of an employee. The NAF also has a well-established system of discussing until a decision is made, but then following it loyally. Good information is the basis for creating the necessary understanding of the change process. For the NAF, it will be relevant to look at the current and future challenges and the assigned mandate given by society. Changes must be made to enable the organization to fulfill its mandate. Experiencing success in the early stages of the change process may increase optimism and personal confidence. The skeptics will be pleased to see evidence of the successful activities. The management can facilitate learning by breaking down complex tasks into smaller pieces that can produce some rapid change effects without costing too much for the individual employee. To experience that the change gives the desired efficiency can increase the implementation effort (Yukl and Lepsinger, 2004). Changing established habits and acquiring new knowledge involves uncertainty. There will be periods of successes and of adversity. The employees will feel powerless, tired and frustrated along the way. Yukl and Lepsinger point out that it is better to inform everyone that change can be difficult for extended periods rather than presenting it as a universal solution that will only be for the better. Then the staff is prepared and better able to take the initiative to solve problems on their own with the support of the management. The gain lies in creating an understanding of the need for change. Being able to highlight gradual successes and encourage change and development is important. This makes it possible to maintain the focus that change is necessary and desirable. The will to be subjected to extra effort may otherwise weaken and move into fatigue and resistance. An important job for management is to persevere and encourage the innovative thinking and optimism (Yukl and Lepsinger, 2004). One way to encourage innovative thinking is by using guidance as an effective communication tool, especially reframing. It is possible to look at obstacles and problems from many perspectives but one may need help to identify new ways of looking at it.

Another guidance technique is asking reflective questions that stimulate people’s problem solving and self-confidence (Boe and Holth, 2017). Yukl and Lepsinger (2004, p. 116) also give four guidelines on how to encourage innovative thinking (see Table 19).

**TABLE 19:** Four guidelines for encouraging innovative thinking.

(1) Encourage people to question assumptions about the work (2) Encourage people to look at problems from different perspectives (3) Encourage people to spend time on developing innovative ideas (4) Provide rewards and recognition for innovative ideas

In the NAF, questioning assumptions about work is especially important in a restructuring process because this is not daily behavior. In order to achieve the greatest gain realization, it may be important to question old tasks before they are transferred into the new education model. On the other hand, it may be useful to question what new issues should be addressed. It is more difficult to get time to develop new ideas.

Yukl and Lepsinger (2004) emphasize that rapid adaptation is more important when the surroundings are turbulent and uncertain. This makes it particularly relevant to the NAF. The NAF is dependent on having the highest competence and the fastest responsiveness, and needs to quickly adapt to new situations and environments. This requires both learning ability and a cognitive openness that allows innovative thoughts. It is perhaps here that the actual cultural change will happen. When the teaching staff is motivated to examine how they can help create the very best learning conditions for the cadets they educate, the will and desire to learn can also be awakened. This requires that the management help to create a safe learning environment where it is allowed to try new paths and make mistakes. The Chief of Defense (Forsvarsstaben, 2018) writes in the NAF’s digitization strategy that it is important to fail quickly and learn from it so that we do not repeat it. Part of the management job in the change process is to ensure the sharing of ideas and new knowledge. As shown in Table 20, Yukl and Lepsinger (2004, p. 118) present five guidelines for facilitating collective learning.

**TABLE 20:** Five guidelines for facilitating collective learning.

Encourage people to experiment systematically with new approaches
Encourage people to find ways to adapt best practices used elsewhere
Encourage the active sharing of ideas and new knowledge to the organization
Encourage the use of after-action reviews to identify lessons learned
Implement systems to facilitate the diffusion of ideas and new knowledge

Although much of this work is about creating a structure where collaboration is prioritized, it is just as much about safeguarding a culture of informal interaction and support. Joint problem solving can help create a joint commitment to the result and an enjoyment of coping. After-action reviews (AAR) are a widely used method in the NAF, but not quite as common in the educational context of the teaching staff. In a transition process where the staff must expect to do their job in a completely new way, it can be of great importance to have a clear direction for how the organization and the individual employee can best learn from experience. The introduction of digitization is not a process that ends; it is a dynamic process that will require the teaching staff to learn to stay abreast of developments in the field. Leadership in this context can include facilitating collaborative learning, where individuals experience positive interdependence to succeed as best as possible, each one being responsible and trustworthy, and having common goals. The leader can stimulate the academic community to use critical thinking which involves asking questions, sorting out information and relating new information to their existing knowledge. Being open to looking at their practice from new perspectives will open up to building a common competence that benefits both the individual and the organization.

Van Velsor et al. (2010, p. 21) defines leadership as a social process that creates direction, alignment and commitment in a collective. These three concepts can make a good contribution to supporting and managing a transformation process that will require a lot of the individual employee.

Primarily, it is all about creating a common idea about what to do (direction), how to do it (interaction) and the willingness to do this (commitment) (Krigsskolen, 2015). A desired end state must be defined and adjusted as the goals are reached so that the development process is maintained and momentum is created. For a digital transformation in education to take place at the Basic Officer Education at the NDUC, such a final state could be continuous adaptation and renewal.

## Conclusion

The theme of the article is the organization’s introduction of digitization in a transition process and its management of this process. We aimed to study if the Norwegian Ministry of Education and Research’s intention of digitization and specific primary goals of learning and teaching from 2017 could be traced in the overarching educational documents in the NAF. We also aimed to investigate whether digitization and any digital tools were mentioned in the NDUC study programs and subject plans for teaching, or if specific goals of digitization were lost in transition from the NAF documents to the NDUC documents. Finally, we also aimed to investigate whether digital tools were implemented in the new NAF Basic Officer Education. The idea was that this could give an indication of how far the organization had come in its work to follow up on the government’s regulations since, from the teacher’s perspective; it immediately appeared to be non-existent.

We first conducted document analyses of the most important documents related to digitization of the university and college sector in Norway, followed by a document analysis of relevant documents issued by the Ministry of Defence, the NAF and the NDUC. We thereafter collected data from key personnel at different levels in the NDUC that were involved with implementing the digitization strategy for the NDUC. The third part of the study consisted of data collection from course evaluations carried out by those who were at the receiving end of the education, namely the cadets enrolled in the new Basic Officer Education.

We conclude that there is an expectation of digitization in the teaching connected to the educational reform in the NAF, but that this it is not communicated systematically enough down through the chain of command. The results from the evaluation show that this process is not well structured. However, there are preliminary plans, and the preparation of the NDUC’s digitization strategy can contribute to a better link between the expectations and what is actually done. The missing link consists of competence enhancement and cultural change in the personnel who are to carry out the teaching. There is no systematic process in the dissemination of expectations regarding digitization of teaching in the educational reform in the NAF, and there is no unified effort from the NDUC down to the Basic Officer Education being conducted at the military academies.

Although we found some significant differences regarding the use of new digital tools in the three different courses, the yielded low effect sizes indicate that these differences are of less practical significance. The differences may merely indicate that the teachers in the three different courses simply decided to use digital tools differently from each other. Another possible explanation is that the teachers in the three different courses looked at their own curriculum and based their use of digital tools upon what they felt was needed in accordance with the curriculum they were supposed to teach in their course.

Demont-Biaggi and Jager (2017) discuss how leaders can remain in control despite the risk that new information technologies can reduce leaders’ control over their decision-making. A challenge here for leaders when implementing digital tools is that the relatively slow decision-making processes does not go well with a rapid technological innovation. The results of this may be friction between different levels of the organization. This friction may arise if levels of command are bypassed because there is an interest in being quick to deal with issues (Alvinius, 2013). This may in turn lead to conflict between the involved parties (Danielsson et al., 2014) and may lead to a poor prioritization of the available resources (Burkle and Hayden, 2001). Our experience through this process has highlighted the importance of the teaching staff being involved in more management perspectives than those directly related to teaching. It could be beneficial if the staff at the university college were informed of upcoming changing requirements so that they could continually be in line with developments in the sector. This can, on the one hand, lead to some worry, but on the other hand it may also activate the human resource at an earlier point in time and support the necessary change and development.

## Possible Limitations and Future Research

A possible limitation is that the participants in our study were quite a homogenous group. An explanation for this is that all our participants have gone through the same type of selection processes when entering into the NAF. This might lead to the absence of any major differences between the participants.

A second possible limitation may be that the questions asked in the course evaluations may suffer from being a bit imprecise, and that there is a lack of explanations for the concepts used in the course evaluations. This might lead to the participants answering without having a clear picture of what they are being asked.

A final possible limitation could be that we conducted a study of a part of an organization that we both are quite familiar with, that is, the NAF. As such, we may have been less objective than researchers from outside the NAF when investigating the same research problem.

New studies within the same topic area might benefit from looking at the management of the process of digitizing the education to create better learning, and thus include the cadet’s perspective.

A possible future venue is to follow up the research conducted in this study with a survey of the first-year cadets to investigate if they see the benefit of getting to know cadets from other branches and meeting physically, or if it would give the same benefit to get to know each through electronic means only. This can be of great importance since the NAF is a collective enterprise where success depends on good interaction and trust in each other’s judgment and competence, and digitization may possibly create greater distance between individuals than desired.

An important requirement for the government’s digitization strategy is research on whether and how this creates higher quality in learning. More research is needed on the connection between digitization and transformation of learning processes.

Even though the cadets are selected young people they do not seem to know what the various digital aids are, or they misinterpret what the questions are asking for. Alternatively, they think that they have used different types of digital tools. We suggest that for the future course evaluations of the Basic Officer Education, short explanations of what different digital tools are, for example, what the e-learning course is, should be included when asking cadets to respond to the questions on digitization.

## Data Availability

The datasets generated for this study are available on request to the corresponding author.

## Ethics Statement

An application was also sent to the Norwegian Social Science Data Service (NSD) to gain approval for the study. The study did not need to be approved by the NSD as we did not collect any personal data. Written informed consent was also not necessary to obtain from the participants, as filling out the course evaluations was part of the current ongoing quality assurance system for the Basic Officer Education.

## Author Contributions

TH and OB designed the study, developed and drew figures and models, wrote and revised the manuscript, analyzed the data, and approved the final version to be published.

## Conflict of Interest Statement

The authors declare that the research was conducted in the absence of any commercial or financial relationships that could be construed as a potential conflict of interest. The reviewer A-KE declared a shared affiliation, with no collaboration, with one of the author, OB, to the handling Editor at the time of the review.
